# Neutropenia induced by high-dose intravenous benzylpenicillin in treating neurosyphilis: Does it really matter?

**DOI:** 10.1371/journal.pntd.0005456

**Published:** 2017-03-13

**Authors:** Rui-Rui Peng, Juan Wu, Wei Zhao, Tengfei Qi, Mei Shi, Zhifang Guan, Haikong Lu, Fuquan Long, Zixiao Gao, Sufang Zhang, Pingyu Zhou

**Affiliations:** Sexually Transmitted Disease Institute, Shanghai Skin Disease Hospital, Shanghai, People's Republic of China; University of Connecticut Health Center, UNITED STATES

## Abstract

**Background:**

Prompt therapy with high-dose intravenous benzylpenicillin for a prolonged period is critical for neurosyphilis patients to avoid irreversible sequelae. However, life-threatening neutropenia has been reported as a complication of prolonged therapy with high doses of benzylpenicillin when treating other diseases. This study aimed to investigate the incidence, presentation, management and prognosis of benzylpenicillin-induced neutropenia in treating neurosyphilis based on a large sample of syphilis patients in Shanghai.

**Methodology/Principal findings:**

Between 1^st^ January 2013 and 31^st^ December 2015, 1367 patients with neurosyphilis were treated with benzylpenicillin, 578 of whom were eligible for recruitment to this study. Among patients without medical co-morbidities, the total incidence of benzylpenicillin-induced neutropenia and severe neutropenia was 2.42% (95% CI: 1.38–4.13%) and 0.35% (95% CI: 0.06–1.39%), respectively. The treatment duration before onset of neutropenia ranged from 10 to 14 days, with a total cumulative dose of between 240 and 324 megaunits of benzylpenicillin. Neutropenia was accompanied by symptoms of chills and fever (5 patients), fatigue (2 patients), cough (1 patient), sore throat (1 patient), diarrhea (1 patient) and erythematous rash (1 patient). The severity of neutropenia was not associated with age, gender or type of neurosyphilis (*p*>0.05). Neutropenia, even when severe, was often tolerated and normalized within one week. A more serious neutropenia did not occur when reinstituting benzylpenicillin in patients with mild or moderate neutropenia nor when ceftriaxone was used three months after patients had previously experienced severe neutropenia.

**Conclusions/Significance:**

Benzylpenicillin-induced neutropenia was uncommon in our cohort of patients. Continuation of therapy was possible with intensive surveillance for those with mild or moderate neutropenia. For severe neutropenia, it is not essential to aggressively use hematopoietic growth factors or broad-spectrum antibiotics for patients in good physical condition after withdrawing anti-neurosyphilis regimen. We did not see an exacerbation of neutropenia in patients with the readministration of benzylpenicillin.

## Author summary

High-dose intravenous benzylpenicillin is an effective treatment for neurosyphilis although it can cause potentially life-threatening drug-induced neutropenia. We investigated the incidence, presentation, management and prognosis of benzylpenicillin-induced neutropenia among neurosyphilis patients treated over a three year period at the Shanghai Skin Disease Hospital. We recruited 578 patients with neurosyphilis who received benzylpenicillin (4 megaunits intravenously every 4 hours for 14 days) according to strict study criteria. For patients without medical co-morbidities, the total incidence of benzylpenicillin-induced neutropenia was 2.42% (95% CI: 1.38–4.13%). The incidence of mild, moderate and severe neutropenia was1.56% (95% CI: 0.76–3.04%), 0.52% (95% CI: 0.13–1.64%), and 0.35% (95% CI: 0.06–1.39%), respectively. The duration of therapy given before the onset of neutropenia ranged from 10 to 14 days, and cumulative doses of benzylpenicillin varied from 240 to 324 megaunits. The accompanying symptoms were tolerated and often normalized within one week under close monitoring of blood counts. Therefore, benzylpenicillin can be continued with surveillance in the presence of mild or moderate neutropenia. Aggressive management is not essential for patients with severe neutropenia in good physical condition after withdrawing anti-neurosyphilis regimen. We did not see an exacerbation of neutropenia in patients with the readministration of benzylpenicillin.

## Introduction

Neutropenia is a condition marked by an absolute neutrophil count (ANC) below 1.5×10^9^/L in adults [1], which can be further categorized as mild (1×10^9^/L≤ANC<1.5×10^9^/L), moderate (0.5×10^9^/L≤ANC<1×10^9^/L) and severe type (ANC<0.5×10^9^/L) [1, 2]. There are many causes including drug-induced neutropenia [2, 3]. Benzylpenicillin-induced neutropenia, a complication of prolonged therapy with high doses, has been well documented when treating infective endocarditis, leading some patients to withdraw necessary treatment and even undergo insidious life-threatening sepsis [4–7].

Syphilis has returned to china with a vengeance in the 21st century [8, 9]. The epidemiology of neurosyphilis (NS) has largely mirrored that of early infective syphilis [10]. Prompt therapy of NS is critical for avoiding irreversible sequelae such as general paresis and tabes dorsalis [11]. The current recommended regimen is high-dose intravenous benzylpenicillin (18 to 24 megaunits daily) for a prolonged period (10 to 14 days) [12, 13]. It is worth considering how to balance the benefit of treating NS with benzylpenicillin and harm if drug-induced neutropenia arises. We analyzed the clinical data of NS patients during three continuous years in order to investigate the incidence, presentation, management and prognosis of benzylpenicillin-induced neutropenia in order to provide helpful experience for other regions with a high burden of syphilis.

## Methods

### Study population and criteria

This retrospective study was approved by the medical ethics committee of the Shanghai Skin Disease Hospital, and conducted according to the principles expressed in the Declaration of Helsinki at the Sexually Transmitted Disease Institute of the Shanghai Skin Disease Hospital from January 1, 2013 to December 31, 2015. We recruited NS patients who (1) underwent their first therapy of high-dose intravenous benzylpenicillin, (2) did not have a recent history of other infections (*e*.*g*.: viral, bacterial, protozoal), (3) denied a past and family medical history of autoimmune diseases, underlying hematological diseases, nutritional deficiencies, splenic sequestration or congenital leukopenia, (4) did not receive chemotherapy, radiotherapy, immunotherapy, oral /intravenous /intramuscular usage of antibiotics, or other new medications in the past three months [14, 15], (5) had no history of alcohol abuse, and (6) had negative HIV status. Patients were excluded if they were under 18 years of age or had a pre-treatment complete blood count (CBC) outside the normal reference range. Written informed consent was obtained before the laboratory test and NS treatment for clinical care and research.

### Case definition and clinical test

NS was defined as having (1) any stage of syphilis, (2) a reactive cerebrospinal fluid-venereal disease research laboratory (CSF-VDRL), and/or (3) an elevated CSF-protein (>50 mg/dL) or pleocytosis (>10 white blood cells/μL) in the absence of other known causes of the abnormalities [12, 13]. Neutropenia was further categorized as mild (1×10^9^/L≤ANC<1.5×10^9^/L), moderate (0.5×10^9^/L≤ANC<1×10^9^/L) and severe (ANC<0.5×10^9^/L) as indicated above [1, 2].

A CBC, urinalysis, routine stool studies for infection and occult blood, biochemical profile, electrolytes, chest radiography and electrocardiograph were performed in all patients before benzylpenicillin therapy. CBC monitoring was performed every other day for mild or moderate neutropenia and every day for severe neutropenia until the value normalized. Other essential tests, including blood culture, sputum culture, biochemical profile, or virus antibody, were also performed when neutropenia occurred. The NS treatment regimen was 4 megaunits of benzylpenicillin as a freshly prepared bolus and slow infusion intravenously every 4 hours for 14 days [12, 13].

### Data extraction and analysis

Clinical data were recorded in terms of age, gender, diagnosis, cumulative dose of benzylpenicillin, days to onset of neutropenia, accompanying symptoms when ANC nadir occurred, clinical management, recovery time and readministration of benzylpenicillin. All data were independently double-coded with Epidata software(version 3.1; Denmark), then transferred into SPSS software (version 18.0; Chicago, IL, USA) for analyses. Descriptive statistics were used to calculate median, percentage, and incidence with 95% confidence interval (CI). A chi-square test (*p*<0.05 indicating statistical significance) was applied to analyze the potential factors associated with neutropenia. The continuous variable "age" was categorized into two subgroups, including age<55 years and age≥55 years. Multivariate logistic regression was used to further identify factors independently associated with neutropenia when significant factors were found by chi-square test.

## Results

**Fig 1:**
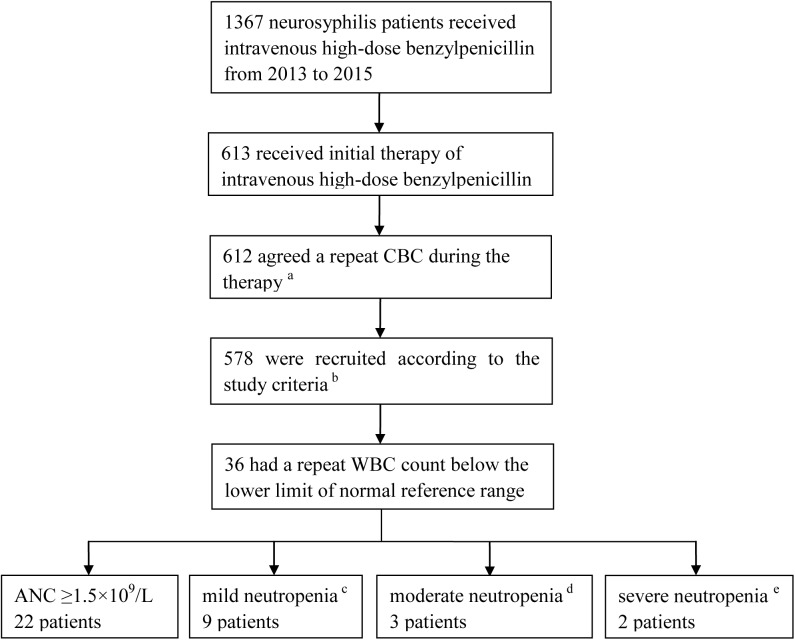
Flowchart of neurosyphilis patients included in the dataset. Abbreviation: CBC, complete blood count; WBC, white blood cell; ANC, absolute neutrophil count. ^a^ One patient did not receive a repeat CBC due to some personal reasons. ^b^ See detailed criteria in the text. ^c^ 1×10^9^/L≤ANC<1.5×10^9^/L ^d^ 0.5×10^9^/L ≤ANC<1×10^9^/L ^e^ ANC<0.5×10^9^/L

A total of 1,367 NS patients were treated with a standard regimen of benzylpenicillin during the study period, 613 of whom received treatment for the first time. Of these, 578 patients underwent repeat CBC during the treatment and were included according to the study criteria. Fourteen patients, all of whom had prior normal CBCs, had a repeat ANC below 1.5×10^9^/L. The median age of these patients was 55 years (range: 27 to 79). Nine were male, and 12 had neurologic complications with a diverse spectrum of diagnoses, including syphilitic meningitis and parenchymatous neurosyphilis. (Fig 1, Tables 1 and 2)

**Table 1:** Characteristics of neurosyphilis patients with and without neutropenia treated with initial therapy of high-dose intravenous benzylpenicillin (n = 578).

Characteristic	No. patients with neutropenia (%) (n = 14)	No. patients without neutropenia (%) (n = 564)
Age (yr), median (range)	55 (27–79)	56 (18–84)
Male	9 (64)	455 (81)
Serum RPR titer, median (range)	1:64 (1:2–1:128)	1:64 (1:2–1:256)
CSF-VDRL titer, median (range)	1:4 (±81:8)	1:4 (negative-1:16)
Elevated CSF-protein ^a^	5 (36)	172 (30)
Pleocytosis ^b^	4 (29)	210 (37)
HIV infected	0 (0)	0 (0)
Symptomatic neurosyphilis	12 (86)	396 (70)

Data are number of patients (%), unless otherwise indicated.

Abbreviation: RPR, rapid plasma reagin; CSF, cerebrospinal fluid; VDRL, venereal disease research laboratory; HIV, human immunodeficiency virus.

^a^ CSF-protein>50 mg/dL.

^b^ CSF-WBC >10white blood cells/μL.

**Table 2:** Summary of neurosyphilis cases with high-dose benzylpenicillin-induced neutropenia.

Case	Age (yr)	Sex	Diagnosis	Days to onset of neutropenia	Cumulative doses (megaunits)	Blood cell counts (×10^9^/L) ^a^				Accompanying symptoms	Blood, throat swab and sputum culture ^b^	Management	Days to recovery of WBC ^c^	Second treatment after 3 months ^d^
						WBC	NEU	RBC	PLT					
1	54	M	general paresis	14	324	0.69	0.04	4.29	85	fever (39.7℃), chills, fatigue mild diarrhea	negative	d/c	4	ceftriaxone, no neutropenia
2	79	M	tabes dorsalis	12	288	3.08	1.12	4.42	143	no	-	c	3	benzylpenicillin, similar neutropenia
3	27	F	asymptomatic neurosyphilis	12	288	3.37	1.17	4.17	106	no	-	c	2	no
4	47	M	tabes dorsalis, otic syphilis	13	312	1.90	0.96	5.02	86	no	-	c	7	ceftriaxone, no neutropenia
5	46	M	general paresis	12	288	2.77	1.34	4.83	117	fever (39℃), chills	negative	c	3	ceftriaxone, no neutropenia
6	66	F	asymptomatic neurosyphilis	12	288	3.54	0.84	3.79	162	no	-	c	6	benzylpenicillin, no neutropenia
7	72	M	syphilitic meningomyelitis	12	288	3.12	0.93	4.59	182	no	-	c	2	no
8	37	M	general paresis	10	240	3.20	1.48	4.44	221	no	-	c	3	benzylpenicillin, no neutropenia
9	57	M	general paresis	10	240	3.69	1.44	4.49	242	fever (38.5℃), chills, cough, sore throat	negative	c	4	no
10	59	F	syphilitic meningitis, ocular syphilis	11	264	3.42	1.49	3.87	125	no	-	c	3	benzylpenicillin, similar neutropenia
11	56	M	tabes dorsalis	11	264	3.33	1.34	4.24	259	no	-	c	2	benzylpenicillin, similar neutropenia
12	63	M	syphilitic meningitis, ocular syphilis, otic syphilis	5	120	2.72	1.13	3.46	161	fever (38.5℃), chills, erythematous rash	negative	d/c	3	ceftriaxone, no neutropenia
13	56	F	syphilitic meningitis	13	312	0.60	0.21	4.22	76	fever (39.3℃), chills, fatigue	negative	d/c	5	ceftriaxone, no neutropenia
14	52	F	tabes dorsalis, ocular syphilis	13	312	2.30	1.07	4.03	128	no	-	c	4	benzylpenicillin, similar neutropenia

Abbreviation: M, male; F, female; WBC, white blood cell; NEU, neutrophil; RBC, red blood cell; PLT, platelet; d/c, discontinued benzylpenicillin; c, continued benzylpenicillin.

^a^ Minimum value recorded during treatment.

^b^ Blood culture was performed in all patients with a recorded oral temperature greater than 38.5℃.Case 9, who also had sore throat and cough, received a throat swab and sputum culture at the same time.

^c^ Days to recovery is the number of days between stopping benzylpenicillin and normalization of ANC.

^d^ The second treatment was either benzylpenicillin (4 million units IV every 4 hours for14 days), or ceftriaxone (1.0 g IV every 12 hours for 15 days).

The total incidence of benzylpenicillin-induced neutropenia was 2.42% (95% CI: 1.38–4.13%, 14/578) among this cohort of patients with NS. Mild neutropenia was observed in 1.56% (95% CI: 0.76–3.04%, 9/578), moderate neutropenia in 0.52% (95% CI: 0.13–1.64%, 3/578), and severe neutropenia in 0.35% (95% CI: 0.06–1.39%, 2/578) of patients. The severity of neutropenia had no association with age, gender or type of neurosyphilis (*p*>0.05). The multivariate logistic regression was not carried out since no significant factors were found by chi-square test.

For the majority (13/14) of patients, the duration of treatment before onset of neutropenia ranged from 10 to 14 days, and the cumulative dose of benzylpenicillin varied from 240 to 324 megaunits. A single patient received 120 megaunits over five days of treatment.The range of nadir total white blood cell (WBC) counts was 0.60 to 3.69×10^9^/L, with nadir ANC from 0.04 to 1.49×10^9^/L. Three patients had concurrent thrombocytopenia. The accompanying symptoms were chills and fever (38.5–39.7℃, 5 patients), fatigue (2 patients), cough (1 patient), sore throat (1 patient), diarrhea (1 patient) and erythematous rash (1 patient). Blood, sputum and throat swab cultures did not reveal an infectious etiology (*e*.*g*.: bacterium and fungus) among the febrile patients. (Table 2)

One patient with syphilitic meningitis, ocular and otic syphilis (case 12) had an itchy rash on the trunk on day 4 and fever (maximum 38.5℃) on day 5. Repeat laboratory testing revealed that his ANC declined to 1.13×10^9^/L. Thus, antihistamine and methylprednisolone (40mg daily) were commenced instead of benzylpenicillin. His symptoms and CBC count normalized on day 8. Subsequently, an alternative regimen of intravenous ceftriaxone (1.0 g every 12 hours for 15 days) (13) was reinstituted uneventfully three months later. (Table 2)

Another two patients (case 1 with general paresis and case 13 with syphilitic meningitis) did well until they had fever, and repeat CBC revealed thrombocytopenia and severe neutropenia (ANC of case 1: 0.04×10^9^/L; ANC of case 13: 0.21×10^9^/L) near the end of therapy. Benzylpenicillin was discontinued. Despite mild symptoms, their fever in the context of a severe neutropenia caused a high level of concern for underlying life-threatening infection, and both patients were transferred to the emergency department. The results of bone marrow examination, Coombs' test, and cytomegalovirus and rubella virus IgM antibodies were not significant. They were given symptomatic relief and supportive treatment rather than human granulocyte colony-stimulating factor, glucocorticosteroid or other prophylactic broad-spectrum antibiotics. Both patients' CBCs returned to normal within four and five days, respectively, after withdrawing benzylpenicillin. Initiation of intravenous ceftriaxone did not induce neutropenia three months later in either patient. (Table 2)

The other 11 patients with mild or moderate neutropenia finished the 14-day therapy with close monitoring of CBC and observation for sequelae of neutropenia. None experienced any severe complication of therapy and all had recovery of a normal ANC within seven days. Some of these patients received a second round of therapy with benzylpenicillin three months later and either had no neutropenia or experienced similar neutropenia without symptoms. (Table 2)

## Discussion

The neutrophil is the most abundant WBC in the peripheral blood and plays a critical role in preventing infections as part of the innate immune system [16]. It has been documented that the offending medications associated with severe neutropenia are methimazole, ticlopidine, clozapine, sulfasalazine, trimethoprim-sulfamethoxazole and dipyrone in descending order of likelihood [17–19].The hematologic complication of hypersensitivity to penicillin is rare, with an overall acute neutropenia of 2.4 to 15.4 cases per million populations over the last 20 years [14]. Based on this large clinical dataset, we concluded a total incidence of 2.42% among healthy NS patients receiving benzylpenicillin. It is noteworthy that the likelihood of acute neutropenia caused by intravenous benzylpenicillin for NS is much higher than that by penicillin for other diseases [14].

Penicillin agents are thought to be able to cause granulopoiesis inhibition [20, 21], and benzylpenicillin-induced neutropenia is dose related more than a pure immunological reaction [15, 22]. As indicated earlier, the duration of beta-lactam therapy prior to the start of neutropenia always exceeded 15 days [21]. We saw cases of neutropenia caused by benzylpenicillin within 14 days probably due to the higher daily dose used for NS than for other diseases. In the 1980s, Al-Hadramy and his colleagues [6] summarized 28 reported cases of benzylpenicillin-induced neutropenia for diseases such as infective endocarditis, bowel obstruction, cellulitis, gangrenous appendix, pneumonia, hemangioma, septic arthritis, and pleural empyema. Therein, 71% patients developed neutropenia after taking 200 megaunits or more, and neutropenia developed in 82% of patients on treatment for two or more weeks, which is consistent with our findings of neutropenia being associated with high-dose and prolonged treatment [6]. Some studies have proposed the hypothesis that genetic and epigenetic modifications predispose an individual to idiosyncratic drug sensitivity [23, 24]. The genetic susceptibility might be associated with an increased risk of neutropenia induced by high-dose benzylpenicillin which needs to be further investigated.

According to previous reports, acute neutropenia was often well tolerated and normalized rapidly [2]. In our study, fever accompanied by general malaise was the first and often the only manifestation in patients. No patients experienced life-threatening complications. Withdrawing benzylpenicillin rapidly led to a recovery in the patient who had an ANC of 0.04×10^9^/L at nadir but no other high-risk symptoms. Even though potential antibody cross-reactivity existed, we found that it was relatively safe when benzylpenicillin was reinstituted in patients with mild or moderate neutropenia, and ceftriaxone in patients with severe neutropenia, three months later.

Previous research has identified that older age (>65 years), septicemia or shock, metabolic disorders such as renal failure, and an ANC under 0.1×10^9^/L were poor prognostic factors associated with drug-induced neutropenia [14]. Thus, in patients with these factors, the empirical use of hematopoietic growth factors, glucocorticosteroid and/or broad-spectrum antibiotics may positively impact the prognosis [18]. Among the 14 NS patients with acute neutropenia in this study, none had metabolic disorders, severe infections, septicemia or septic shock. No patients were given hematopoietic growth factors or broad-spectrum antibiotics, even though three patients were older than 65 years, and one patient had an ANC of 0.04×10^9^/L. We also found the severity of neutropenia had no significant association with age, gender or the type of NS.

Syphilis is far from eradicated, especially in the resource-limited areas worldwide, and it can affect any part of the neuraxis at any stage of infection [25, 26]. There is a growing consensus that NS patients can benefit from regular benzylpenicillin therapy, and high-dose benzylpenicillin is of proven efficacy at the early stage of NS [27]. Here, we outlined benzylpenicillin-induced neutropenia as a complication of NS treatment. Some limitations should be acknowledged. First, due to limited published data on when to obtain surveillance CBCs during treatment, we arranged the first repeat CBC on day 10 unless any clinical symptom occurred beforehand. Thus, asymptomatic neutropenia may have been present in the two patients (case 8 and 9) earlier than day 10. Second, prompt NS therapy was limited to patients whose other medical conditions (*e*.*g*. uncontrolled hypertension or diabetes) were stable in order to minimize risk of therapy. Meanwhile, HIV co-infected patients were not included in the analysis because of possible confounding of leukopenia caused by HIV. These factors might limit the generalizability of our findings.

In conclusion, benzylpenicillin-induced neutropenia was well tolerated in our cohort of patients with mild or moderate type. It also normalized rapidly without aggressive management for those with severe neutropenia after withdrawing anti-neurosyphilis regimen. We did not see an exacerbation of neutropenia in patients with the readministration of benzylpenicillin.

## References

[1] Colledge NR, Walker BR, Ralston SH. Davidson's principles and practice of Medicine. 21 ed. Craig JIO, McClelland DBL, Watson HG, editors. Edinburgh: Churchill livingstone; 2010 P. 1000–1

[2] Boxer LA. How to approach neutropenia. Hematology Am Soc Hematol Educ Program. 2012;2012:174–82. 10.1182/asheducation-2012.1.174 23233578

[3] Navarro-Martinez R, Chover-Sierra E, Cauli O. Non-chemotherapy drug-induced agranulocytosis in a tertiary hospital. Hum Exp Toxicol. 2016 3;35(3):244–50. 10.1177/0960327115580603 25845587

[4] Colvin B, Rogers M, Layton C. Benzylpenicillin-induced leucopenia. Complication of treatment of bacterial endocarditis. Br Heart J. 1974 2;36(2):216–9. 4818153 10.1136/hrt.36.2.216 PMC458820

[5] Snavely SR, Helzberg JH, Bodensteiner DC, Plapp FV, Davis JW, Hodges GR. Profound neutropenia associated with benzylpenicillin. South Med J. 1983 10;76(10):1299–302. 6623146 10.1097/00007611-198310000-00026

[6] Al-Hadramy MS, Aman H, Omer A, Khan MA. Benzylpenicillin-induced neutropenia. J Antimicrob Chemother. 1986 2;17(2):251–3. 3700289 10.1093/jac/17.2.251

[7] Walz B, Zupnick J, Uden D, Keating A. Penicillin-induced agranulocytosis. West J Med. 1988 10;149(4):460–2. 3227691 PMC1026506

[8] Chen ZQ, Zhang GC, Gong XD, Lin C, Gao X, Liang GJ, et al Syphilis in China: results of a national surveillance programme. Lancet. 2007 1 13;369(9556):132–8. 10.1016/S0140-6736(07)60074-9 17223476 PMC7138057

[9] Tucker JD, Chen XS, Peeling RW. Syphilis and social upheaval in China. N Engl J Med. 2010 5 6;362(18):1658–61. 10.1056/NEJMp0911149 20445179

[10] Shi M, Peng RR, Gao Z, Zhang S, Lu H, Guan Z, et al Risk profiles of neurosyphilis in HIV-negative patients with primary, secondary and latent syphilis: implications for clinical intervention. J Eur Acad Dermatol Venereol. 2016 4;30(4):659–66. 10.1111/jdv.13514 26660338

[11] Zhou P, Gu X, Lu H, Guan Z, Qian Y. Re-evaluation of serological criteria for early syphilis treatment efficacy: progression to neurosyphilis despite therapy. Sex Transm Infect. 2012 8;88(5):342–5. 10.1136/sextrans-2011-050247 22363023

[12] Workowski KA, Bolan GA. Sexually transmitted diseases treatment guidelines, 2015. MMWR Recomm Rep. 2015 6 5;64(RR-03):1–137. 26042815 PMC5885289

[13] STD Association, Chian CDC. The diagnosis and treatment guidelines of syphilis, gonorrhoea, genital herpes and chlamydial trachomatis infection (2014). Chin J Dermatol. 2014;47(5):365–72. Chinese.

[14] Andres E, Zimmer J, Affenberger S, Federici L, Alt M, Maloisel F. Idiosyncratic drug-induced agranulocytosis: Update of an old disorder. Eur J Intern Med. 2006 12;17(8):529–35. 10.1016/j.ejim.2006.07.012 17142169

[15] Andres E, Maloisel F. Idiosyncratic drug-induced agranulocytosis or acute neutropenia. Curr Opin Hematol. 2008 1;15(1):15–21. 10.1097/MOH.0b013e3282f15fb9 18043241

[16] Curtis BR. Drug-induced immune neutropenia/agranulocytosis. Immunohematology. 2014;30(2):95–101. 25247619

[17] Andersohn F, Konzen C, Garbe E. Systematic review: agranulocytosis induced by nonchemotherapy drugs. Ann Intern Med. 2007 5 1;146(9):657–65. 17470834 10.7326/0003-4819-146-9-200705010-00009

[18] Andres E, Zimmer J, Mecili M, Weitten T, Alt M, Maloisel F. Clinical presentation and management of drug-induced agranulocytosis. Expert Rev Hematol. 2011 4;4(2):143–51. 10.1586/ehm.11.12 21495924

[19] Pick AM, Nystrom KK. Nonchemotherapy drug-induced neutropenia and agranulocytosis: could medications be the culprit? J Pharm Pract. 2014 10;27(5):447–52. 10.1177/0897190014546115 25124379

[20] Neftel KA, Hauser SP, Muller MR. Inhibition of granulopoiesis in vivo and in vitro by beta-lactam antibiotics. J Infect Dis. 1985 7;152(1):90–8. 4008995 10.1093/infdis/152.1.90

[21] Scheetz MH, McKoy JM, Parada JP, Djulbegovic B, Raisch DW, Yarnold PR, et al Systematic review of piperacillin-induced neutropenia. Drug Saf. 2007;30(4):295–306. 17408306 10.2165/00002018-200730040-00002

[22] Munshi HG, Montgomery RB. Severe neutropenia: a diagnostic approach. West J Med. 2000 4;172(4):248–52. 10778379 10.1136/ewjm.172.4.248 PMC1070837

[23] Tesfa D, Keisu M, Palmblad J. Idiosyncratic drug-induced agranulocytosis: possible mechanisms and management. Am J Hematol. 2009 7;84(7):428–34. 10.1002/ajh.21433 19459150

[24] Johnston A, Uetrecht J. Current understanding of the mechanisms of idiosyncratic drug-induced agranulocytosis. Expert Opin Drug Metab Toxicol. 2015 2;11(2):243–57. 10.1517/17425255.2015.985649 25424130

[25] Berger JR, Dean D. Neurosyphilis. Handb Clin Neurol. 2014;121:1461–72. 10.1016/B978-0-7020-4088-7.00098-5 24365430

[26] Chao JR, Khurana RN, Fawzi AA, Reddy HS, Rao NA. Syphilis: reemergence of an old adversary. Ophthalmology. 2006 11;113(11):2074–9. 10.1016/j.ophtha.2006.05.048 16935333

[27] Musher DM. Neurosyphilis: diagnosis and response to treatment. Clin Infect Dis. 2008 10 1;47(7):900–2. 10.1086/591535 18715158

